# A Bibliometric Analysis of the Global Trend of Using Alginate, Gelatine, and Hydroxyapatite for Bone Tissue Regeneration Applications

**DOI:** 10.3390/polym13040647

**Published:** 2021-02-22

**Authors:** Mohamed Saiful Firdaus Hussin, Aludin Mohd Serah, Khairul Azri Azlan, Hasan Zuhudi Abdullah, Maizlinda Izwana Idris, Ihwan Ghazali, Amir Husni Mohd Shariff, Nurul Huda, Azrul Abidin Zakaria

**Affiliations:** 1 Faculty of Mechanical and Manufacturing Engineering Technology, Universiti Teknikal Malaysia Melaka, Durian Tunggal, Melaka 76100, Malaysia; aludin@utem.edu.my (A.M.S.); khairul.azri@utem.edu.my (K.A.A.); ihwan@utem.edu.my (I.G.); 2 Faculty of Mechanical and Manufacturing Engineering, Universiti Tun Hussein Onn Malaysia, Batu Pahat, Johor 86400, Malaysia; hasan@uthm.edu.my (H.Z.A.); izwana@uthm.edu.my (M.I.I.); 3 Faculty of Food Science and Nutrition, Universiti Malaysia Sabah, Kota Kinabalu, Sabah 88400, Malaysia; amirhusni@ums.edu.my; 4 Department of Mechanical Engineering, Universiti Tenaga Nasional, Kajang, Selangor 43000, Malaysia; aazrul@uniten.edu.my

**Keywords:** bone tissue, scaffold, alginate, gelatine, hydroxyapatite

## Abstract

Collecting information from previous investigations and expressing it in a scientometrics study can be a priceless guide to getting a complete overview of a specific research area. The aim of this study is to explore the interrelated connection between alginate, gelatine, and hydroxyapatite within the scope of bone tissue and scaffold. A review of traditional literature with data mining procedures using bibliometric analyses was considered to identify the evolution of the selected research area between 2009 and 2019. Bibliometric methods and knowledge visualization technologies were implemented to investigate diverse publications based on the following indicators: year of publication, document type, language, country, institution, author, journal, keyword, and number of citations. An analysis using a bibliometric study found that 7446 papers were located with the keywords “bone tissue” and “scaffold”, and 1767 (alginate), 185 (gelatine), 5658 (hydroxyapatite) papers with those specific sub keywords. The number of publications that relate to “tissue engineering” and bone more than doubled between 2009 (1352) and 2019 (2839). China, the United States and India are the most productive countries, while Sichuan University and the Chinese Academy of Science from China are the most important institutions related to bone tissue scaffold. Materials Science and Engineering C is the most productive journal, followed by the Journal of Biomedical Materials Research Part A. This paper is a starting point, providing the first bibliometric analysis study of bone tissue and scaffold considering alginate, gelatine and hydroxyapatite. A bibliometric analysis would greatly assist in giving a scientific insight to support desired future research work, not only associated with bone tissue engineering applications. It is expected that the analysis of alginate, gelatine and hydroxyapatite in terms of 3D bioprinting, clinical outcomes, scaffold architecture, and the regenerative medicine approach will enhance the research into bone tissue engineering in the near future. Continued studies into these research fields are highly recommended.

## 1. Introduction

**Figure 1:**
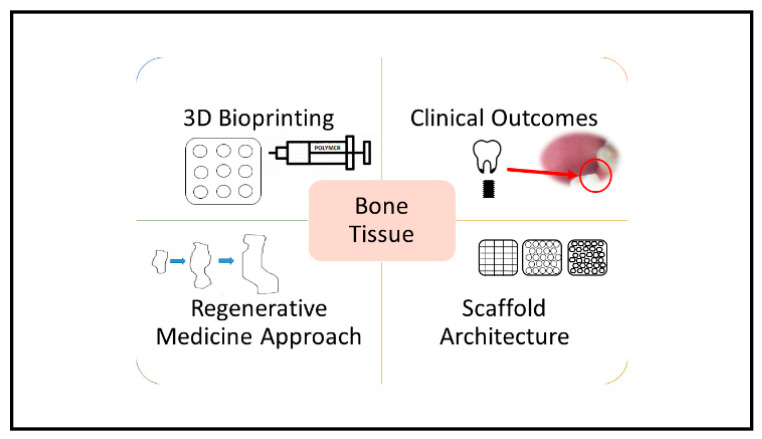
Current trend of research focuses concerning bone tissue engineering.

**Figure 2:**
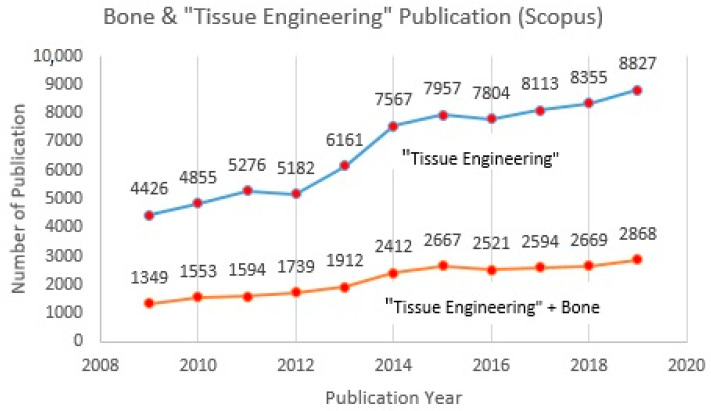
Published articles for tissue engineering and bone in Scopus database (2009–2019).

Tissue engineering or tissue regeneration represents a combination of biomaterials, biological signals, and cells, considering their biocompatibility, bioactivity, strength, manufacturability, and functional suitability [1,2,3,4,5]. 3D bioprinting, clinical outcomes, scaffold architecture, and regenerative medicine approach are among the main issues highlighted and which draw the attention of researchers at present (Figure 1). The driving factors that stimulate the growing demand in tissue engineering research are faster healing processes, tissue repair, and chronic diseases [6,7,8,9,10]. A consistently increasing trend for publications in tissue engineering research over the past ten years also reflects the expansion of worldwide interest in this issue (Figure 2). Cartilage, bone, skin, tooth, cardiac, and vascularization are the most popular topics discussed regarding tissue regeneration [11,12,13,14,15,16]. According to the Scopus database, the number of publications that relate to “tissue engineering” and bone increased more than two-fold between 2009 (1352) and 2019 (2868) (Figure 2). Cartilage is considered a soft, elastic tissue and it appears when flexibility is required. Meanwhile, bone is considered a hard, rigid tissue and it serves as a source of calcium that withstands deformation. By nature, cartilage will reduce the impact when bones collide with each other.

The trend from Figure 2 indicates that research in the “tissue engineering” field, especially studies related to bone, is still developing and attracting increased attention from scientists and the academic community. At this point, tissue engineering has become a well-known topic in recent years, especially with regard to biomaterials. Natural polymers and ceramics are among the most common materials discussed for tissue regeneration currently, due to their abundant naturally, low-cost, non-toxicity, and compatibility with the applications. A combination of polymers and ceramics is believed to be the best way to merge artificial materials for bone tissue [17,18,19,20,21,22]. Although different types of biomaterial are compatible with tissue engineering applications, only alginate, gelatine, and hydroxyapatite will be discussed in this study. An investigation of published articles in the Scopus database revealed that as keywords combined, the number of publications decreased. In the case of searching articles with the combined keywords “alginate, gelatine, hydroxyapatite, scaffold, and bone tissue”, only 2 articles were found and both had been published between 2009 and 2019 (Table 1). Thus, it will be a relevant strategy to penetrate into less-explored knowledge about the topic that has received increased attention over the last decade. Furthermore, high concentration alginate is believed to have the capability of improving the mechanical strength of scaffold [23]. G-blocks of alginate are able to participate in intermolecular cross-linking with divalent cations, normally a positive cation, for instance, calcium, to form hydrogels. The composition, sequence, G-block length, and molecular weight are thus critical factors affecting the physical properties and strength of alginate and scaffold [24]. However, alginate lacks efficient sites for cell adhesion. In this case, gelatine and hydroxyapatite improve the properties of alginate by producing cells with good viability, a good proliferation rate, and adhesion, as well as encapsulation behavior. In a sense, alginate will boost the strength of a scaffold structure [25,26].

## 2. Alginate, Gelatine, and Hydroxyapatite for Bone Tissue Regeneration

A number of publications were issued, discussing the materials’ extraction, scaffold preparation process, and cell culture testing (in vitro, ex vivo, and in vivo) of different combinations of materials, which resulted in important discoveries for bone tissue engineering.

### 2.1. Alginate

Alginate has been extensively used in various fields, particularly in tissue regeneration, for its ease of gelation, low toxicity, low price, abundant availability, and biocompatiblility [27]. Nevertheless, it also has low bioactivity properties [18,28]. Solid form alginate can be produced through either the calcium alginate process or the alginic acid process. Different alginate sources provide different types of chemical structures and these will affect the mechanical and physical properties. The moisture from alginate dressings is capable of accelerating the progress of wound healing [29]. Chee et al. stated that alginate can be extracted using a hot method and a cold method [30]. From another viewpoint, Leal et al. [31] and Youssouf et al. [32] used an ultrasound-assisted extraction method, which had the advantage of reducing alginate extraction time.

### 2.2. Gelatine

Gelatine is a biopolymer derived from collagen that supports the structure of an animal or human body. It is naturally abundant, low-cost, biodegradable, biocompatible, and has low antigenicity properties. Most importantly, there are a high number of functional groups that enable structure modification. Hoque et al. [33] described gelatine as a multi-purpose biopolymer suitable for inclusion as scaffolding material for tissue engineering. Further, Karim and Bhat [34] and Herpandi et al. [35] highlighted in their studies the possibility of extracting gelatine from various fish by-products as a possible option which would avoid the issue of being non-halal. It can be collected using a heat treatment process on fish by-products that are pre-treated with acid or alkaline [36]. Meanwhile, Abedinia et al. observed that duck feet gelatine film are suitable for use as a good alternative material to bovine gelatine film [37].

### 2.3. Hydroxyapatite

As for hydroxyapatite, it is capable of supporting bone growth given its similar properties to hard tissue and its ability to acculturate with the surrounding tissues. It has also been regarded as one of the most valuable material in bone tissue engineering for assisting cell adhesion and, proliferation, and improving a structure’s mechanical strength [38]. Granito et al. [39] and Pon-On et al. [40] outlined the possibility of extracting hydroxyapatite using a calcination method or alkaline hydrolysis. Other than hydroxyapatite, tricalcium-phosphate can be considered as a substitute due to its excellent biodegradability and it can be dissolved in a shorter time [41,42].

### 2.4. Scaffold Preparation

The scaffold preparation for bone tissue depends on factors such as osteogenic differentiation, cell proliferation, cell attachment and viability, vascularization, and host integration [43]. Several manufacturing techniques are used including mold pressing [44], solvent casting [45,46], salt leaching [47,48], emulsion coating [49], polymer foam replication [50,51], the cryogelation technique [52,53,54], the polymer sponge method [55], freeze-drying [56,57,58], electrospinning [59,60,61], and 3D bioprinting [62,63,64,65].

### 2.5. Current Studies Related to Alginate, Gelatine, and Hydroxyapatite

Daniela et al. concluded that alginate hydrogel is capable of delivering mesenchymal stromal cell (MSC) and recruiting endogenous cells. However, osteogenic stimuli are needed as a supplement to regenerate critical-sized segmental femoral defects [66]. Ramaswamy et al. [67] found in their study that surface topography using a microcasting technique encouraged cell cultures on hydroxyapatite. Using a pillar and isolated island topographies initiated a new possibility of patterning the scaffold inspired by nature. Kruppke et al. observed in their experiment that gelatine modified monetite is biocompatible as a bone tissue substitution for human osteoblasts [68].

Mahmoud et al. [69] observed that a combination of porous scaffold with alginate coating shows positive results for both in vitro studies with simulated body fluid and in vivo experiments using the femur bone of a rat, where the calcium phosphate ratio of regenerated bone is equal to the standard rat bone. Meanwhile, research done on rat bone marrow mesenchymal stem cells using a bioglass/gelatine/alginate scaffold [70] reported that the presence of bioglass improved the compressive strength and biomineralization, as well as cell adhesion and biocompatibility. From [69] and [70], it was proven that alginate and bioglass are capable of enhancing the mechanical strength of hydroxyapatite and alginate scaffolds, respectively. Ho et al., in their work, observed that Injectable sodium alginate/beta-tricalcium phosphate (SO3T20) microspheres are biocompatible for bone regeneration and give no adverse reaction; they are also encouraging for osteogenesis [71]. Przekora et al. suggested human bone explant as an osseointegration testing model since it could remain alive under in vitro conditions for approximately 50 days [72]. Fenghua et al., in their work, found that carboxymethyl chitosan/sodium alginate was completely halted in the micron-fibers. The scaffold shows excellent tensile strength and, no significant cytoxicity, and it promotes osteoblast adhesion [73]. Reakasame et al. investigated the effect of bioglass microparticles on fabrication and physicochemical properties of alginate dialdehyde-gelatine hydrogel. The existence of bioactive glass decreases the degradation rate and enhances bioactivity. The viability of MG-63 cells increases in the first week of cultivation. Cell viability is better without bioactive glass [74]. Zhao et al., in their work, observed that the pore size, porosity, water absorption and degradation rates of silver nanoparticle-gelatin/alginate (AgNP–Gel/Alg) scaffolds increased compared to Gel/Alg scaffolds. Cell proliferation activity in the 200 μM group was remarkably higher than in the control group [75].

Benedini et al. [76] filled alginate/hydroxyapatite in their study with ciprofloxacin drugs to evaluate the antibacterial properties of the composite. It turned out that a drug-loaded composite shows good bioactivity and high biocompatibility with calvaria rat osteoblast, as well as antibacterial activities against osteomyelitis. Abouzeid et al. found that polyvinyl alcohol-grafted cellulose nanofiber with sodium alginate (PVA/BF-CNF/SA) scaffold with nano-DCPDH can be an excellent substance for bone tissue research [77]. A combination of PGA/PLLA/HAP was capable of improving scaffold hydrophilicity. The degradation rate of PGA increased the contact area between PLLA and bodily fluids to provide a suitable environment for osteoblastic growth and proliferation [78]. A natural and synthetic polymer combination with hydroxyapatite has shown good viability and excellent proliferation of human osteoblast cells [79]. Meanwhile, an in vitro study using dual-doped hydroxyapatite coated with Ti-6AL-4 V has shown excellent biocompatibility towards the cell line [80]. A tomographical and histological study showed that the regeneration of critical-sized calvarial bone defects in vivo at the 28th day after an implantation of MSC-seeded PHB/HA/ALG/MSC scaffolds is 3.6 times higher than the formation of bone tissue at 22–28 days, in comparison with acellular PHB/HA/ALG scaffolds [81]. Lima et al. [82] reported an injectable substitute mixture of hydroxyapatite and beta-tricalcium phosphate exhibited no cytotoxicity and excellent results from in vivo, using tibia bone defects in rabbits at 30 and 60 days.

Sharmila et al. analyzed the usage of plant-based scaffolds in their work and found that Alg/CMC/SO scaffold presented higher cell viability than Alg/CMC/SO-CQ scaffold, which gave better cellular biocompatibility. Further investigations on plant-based Alg/CMC/SO scaffold as a potential biopolymer scaffold for bone tissue regeneration is highly recommended [83]. Deng et al. summarized that plant-based loaded scaffolds enhance the proliferation of bone marrow stromal cells (rBMSCs) and migration, and the tubule formation of human umbilical vein endothelial cells (HUVECs). A high concentration of hydroxy-saflower yellow A (HYSA)/scaffolds has a notably better capability to assist new bone formation than undoped scaffolds at eight weeks’ post-surgery [84].

## 3. Bibliometric Analysis on Scopus Database

This article aims to find international direction on biomaterial studies for bone tissue engineering in terms of biopolymers and bioceramics, specifically alginate, gelatine, and hydroxyapatite. Data for this purpose were collected from the Scopus database covering the last decade, starting in 2009. Published studies were explored using a search strategy to scrutinize trends. There are two primary aims of the authors of this study: (1) To discover the direction steering the related research in the field of bone tissue engineering using scaffold, especially using alginate, gelatine and hydroxyapatite. (2) To perform a novel study, which aims to enhance cell bioactivity, biocompatibility, viability and antibacterial activity, based on obtained data. The following terms establish the pillar of the bibliometric analysis: number of publications, type of publication, leading journals, authors, institutions, and countries. A bibliometric analysis has previously been defined as “the application of mathematical and statistical methods to books and other media of communications” [85]. Nevertheless, there are a few constraints connected with bibliometric data [86,87].

- Bibliometric data cannot be interpreted as a holistic response to quality measurement. For example, the number of citations of an article does not necessarily mean that it is of high quality, but symbolizes its impact or usefulness.
- In publications, not only English language articles are published but also many different languages.
- A bibliometric analysis does not include whole research areas and does not index all publications.
- The number of citations is highly dissimilar between disciplines. So, a direct comparison cannot be made using it.

In the initial step of the bibliometric literature analysis, the Scopus database was adopted as the data source of this research. The Scopus web site claims “…the largest abstract and citation database of peer-reviewed literature: open access articles (more than 8.5 million), books (more than 194,000) and conference proceedings (more than 9 million)”. Publications on biomaterial (alginate, gelatine, and hydroxyapatite) and bone tissue were prudently searched for and retrieved from the Scopus database. A bibliometric literature analysis was carried out using important factors such as descriptive (year of publication, subject categories, journal counts), relational (collaborations among authors, countries, institutions) and qualitative (citations, impact factors) terms, whose titles included the following main keywords: “bone tissue, scaffold”, and sub-keywords: “alginate, gelatine, hydroxyapatite”. If publications contained these keywords in the title or abstract, the keywords were retrieved for further analysis. The data were retrieved in December 2020 and included a time span from 2009 to 2019. The query of the search was TITLE-ABS-KEY (“bone tissue” AND scaffold) AND PUBYEAR >2008 and PUBYEAR <2020. The Pajek and Vosviewer software packages [88,89] were used in the social network analysis for the visualization of networks. After retrieving the raw data (CSV Format) from Scopus, data were converted into .xls format before the analysis was carried out. Excel was developed by Microsoft and it is a great tool with various features for helping with a bibliometric analysis.

## 4. Global Trend

**Figure 3:**
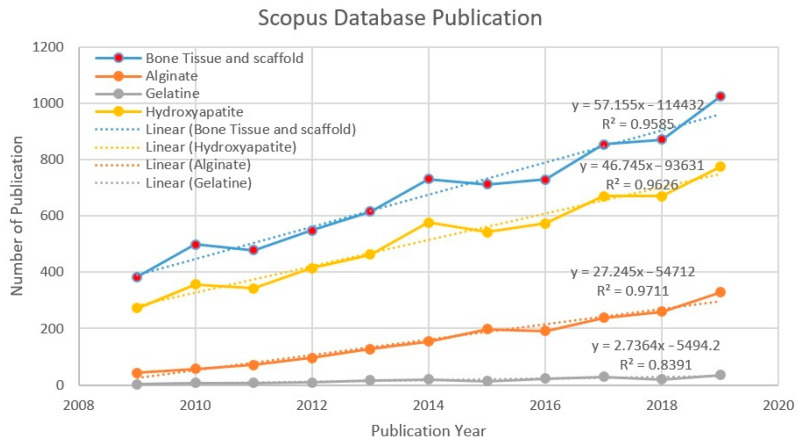
Number of publications per year (2009–2019).

Figure 3 shows the trend in scientific publications between 2009 and 2019. The number of publications has increased each year. It shows that alginate, gelatine, and hydroxyapatite, in terms of “bone tissue” and scaffold, are worthwhile and relevant topics to be further discussed. Data mining and analysis from the Scopus database using the keywords “bone tissue” and scaffold shows that 7446 publications on scaffold and bone tissue were published. The trend continues to increase every year. The number of publications related to bone tissue and scaffold grew by 90.86% between 2009 and 2014, and by 40.08% from 2014 to 2019, respectively. A more specific search was executed to find more precise data within the “bone tissue” and scaffold publications (7446) in the database. 1767 publications for the “alginate” keyword, 185 publications for the “gelatine” keyword, and 5658 publications for the “hydroxyapatite” keyword were found. Figure 3 appears to show a linear increase, giving an idea of the continuous growth rate of publications. The relationship between x and y was y = 57.155x – 114,432 (R^2^ = 0.9585) for bone tissue and scaffold, y = 46.745x – 93,631 (R^2^ = 0.9626) for hydroxyapatite, y = 27.245x – 54,712 (R^2^ = 0.9711) for alginate, and y = 2.7364x − 5494.2 (R^2^ = 0.8391) for gelatine. A coefficient of the determination values of each search indicates the linear regression line was highly consistent with the actual results, which show continuously increasing numbers of publications.

**Figure 4:**
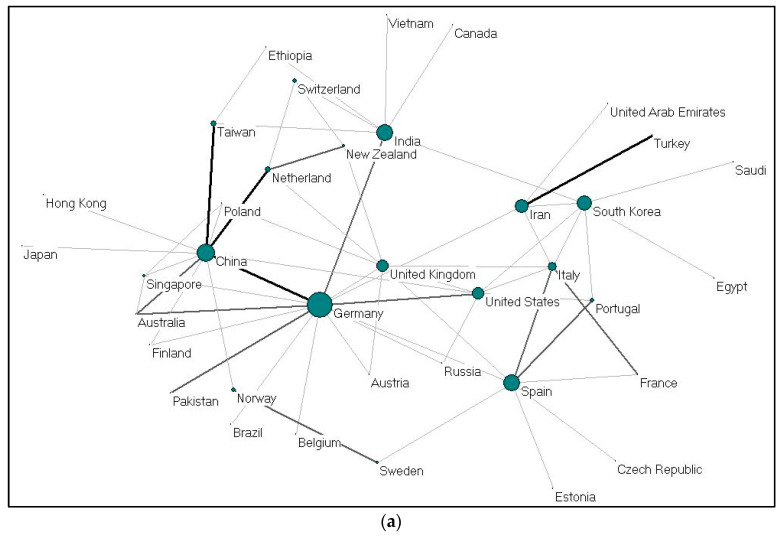

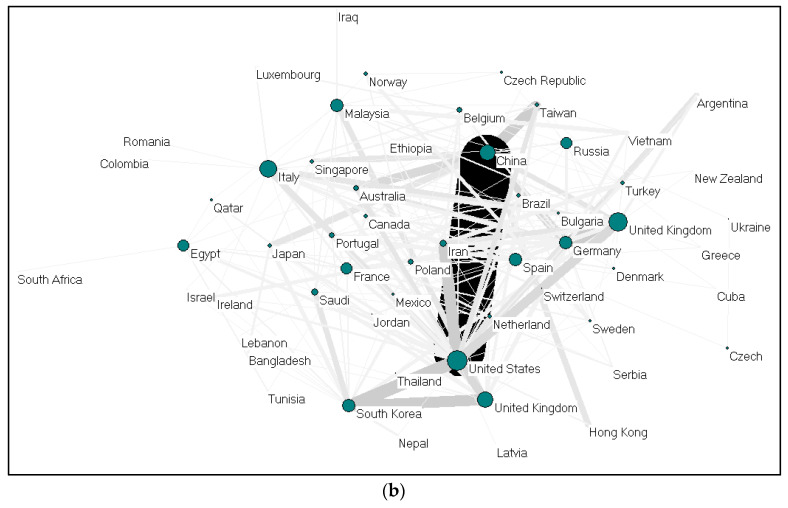
(**a**) Collaboration between countries on gelatine (2009–2019); (**b**). Collaboration between countries on alginate (2009–2019).

The type of publication is considered one of the important parts of a bibliometric analysis carried out for scientific publications. From an academic point of view, the type of publication is an important criterion for both academic advancement and publication incentives. More than 70% of the publications have been published as articles for all keywords (Figure 4). 1329 articles with ‘alginate’, 135 articles with ‘gelatine’, and 4526 articles with ‘hydroxyapatite’ as a keyword were discovered. Reviews are the next most common form of publication with a total number of 830, followed by proceedings (388 publications). The term ‘other’ refers to a conference review, editorial, erratum, note, short survey, or data paper (Table 2).

Table 3 shows the most participative and productive countries, according to the number of publications. China dominates, with first ranking for alginate, gelatine and hydroxyapatite with 511 publications (28.92%) related to alginate with 9166 total citations, 40 publications (21.62%) related to gelatine, and 1617 (28.58%) publications related to hydroxyapatite between 2009 and 2019. The next most participative countries are the United States and India, with 338 publication (13,097 citations), and 181 publications (5208 citations) for alginate, respectively; and Germany and Italy, with 26 publications (863 citations), and 21 publications (512 citations) for gelatine, respectively. Meanwhile, the United States and South Korea published the most after China concerning hydroxyapatite, with 973 publications (26,727 citations), and 423 publications (9621 citations) each. Despite producing fewer publications for alginate (114), gelatine (26), and hydroxyapatite (349), Germany received a total of 5067, 863 and 10,273 citations for these publications, more than the majority of the countries when compared to the number of publications. To some extent, Germany dominates the h-index by occupying the top three positions for all keywords. Results indicate that these publications are of high quality. The countries with the highest h-index are the United States, China, and Germany for alginate; Germany, China and Italy for gelatine; and again, the United States, China and Germany for hydroxyapatite.

Collaboration between countries on gelatine (37 nations) and alginate (58 nations) were visualized using Pajek, as shown in Figure 4a,b. Each country is presented as a node, and the size of the nodes is proportional to the total number of collaboration; the thickness demonstrates the strength of collaboration. China-United States, United Kingdom-Germany, and United States-South Korea collaborations ranked first for alginate. For gelatine, cooperations between Iran-Turkey, China-Germany, China-Taiwan and China-Netherlands ranked the highest. The cooperation network between EU countries seems to be very dense. Meanwhile, Germany and China (gelatine); and the United States and China (alginate) act as countries that build cooperation networks among other countries.

Authors who publish the most on all the topics are Boccaccini Aldo Roberto with 50, 11 and 111 publications for alginate, gelatine, and hydroxyapatite, respectively (Table 4). However, the author with the most citations is Ramakrishna Seeram. The total number of publications (59) suggests that publications other than alginate, gelatine, and hydroxyapatite contributed the most to the h-index for Ramakrishna Seeram. It should be noted that Boccaccini Aldo Roberto and Roether Judith have co-authored a large number of papers. Ramakrishna Seeram and Xu Hockin are considered the most productive authors, considering their average citations per article are 63.74 and 61.23 respectively.

**Figure 5:**
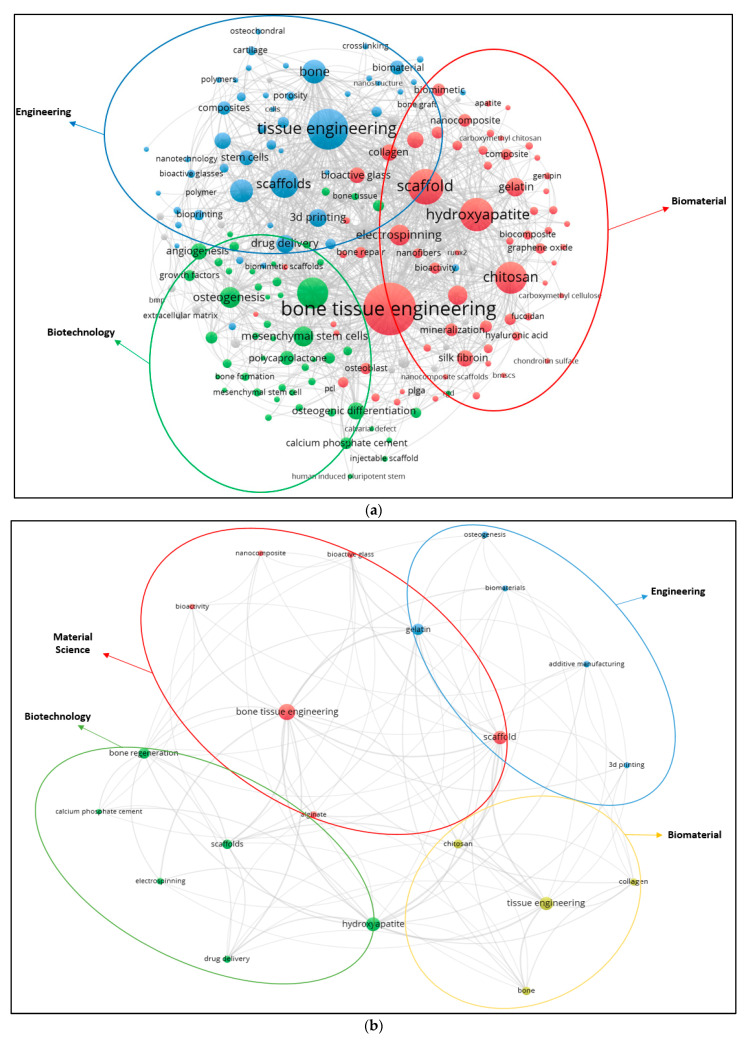

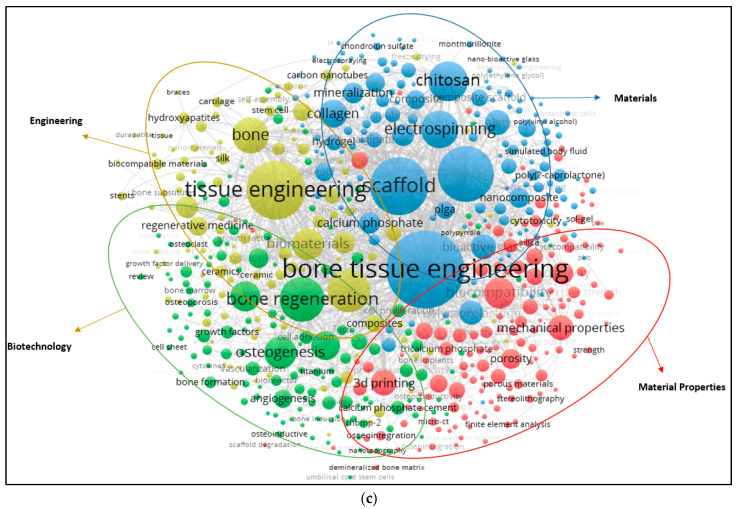
(**a**) Author keywords map for alginate (2009–2019); (**b**) Author keywords map for gelatine (2009–2019); (**c**) Author keywords map for hydroxyapatite (2009–2019).

The top 15 words used in the paper titles are summarized in Table 5. During the period between 2009 and 2019, the most preferred word in publication titles for alginate and hydroxyapatite were ‘‘bone” (1189) and (3655) and ‘‘scaffold” (104) for gelatine. The co-occurrence relationships among the most frequently used keywords in publications are also visualized (Figure 5a–c). A keyword analysis of research papers in certain areas is very useful to predict ongoing and future trends in the science and engineering branches. A keyword analysis was carried out with author keywords in the field of alginate, gelatine and hydroxyapatite. A total of 2683, 471 and 6841 different keywords were identified from 2009 to 2019 in the field of alginate, gelatine, and hydroxyapatite. The minimum number of co-occurrence set was five times. Figure 5 shows that either three or four main clusters are characterized by the most commonly used keywords in the alginate, gelatine and hydroxyapatite area, respectively.

Table 6 presents the top ten publishing journals. Three exceptional journals are: Materials Science and Engineering C with 89 publications, the Journal of Biomedical Materials Research Part A with 79 publications, and Acta Biomaterialia with 62 publications for alginate; respectively, the journals produced 12, 11, and 7 publications for gelatine; and 288, 267 and 198 publications each for hydroxyapatite. Materials Science and Engineering C is the journal, which has by far the largest number of publications on all keywords. According to the cite score analysis for 2019, Materials Science and Engineering C, Acta Biomaterialia, Biomaterials, and Carbohydrate Polymer scored above ten. Over 94.14% of the publications were published in English, while only 5.09% were published in Chinese. The remainder consisted of publications written in Korean, Russian, and Spanish, representing less than 1% each. Table 7 shows the top ten institutes’ statistical information based on the number of papers according to selected keywords. Among the top ten institutes, half originate from China. This is followed by Germany, Iran, Singapore, and Portugal. Sichuan University and the Chinese Academy of Science, China are the most important institutions related to bone tissue scaffold.

## 5. Conclusions and Final Considerations

The current scientometrics analysis makes several important contributions to bone tissue scaffold material using alginate, gelatine and hydroxyapatite subjects, based on a bibliometric study. The findings attained from the bibliometric analysis provide insights for future research. According to the raw data from the Scopus database, publication characteristics such as quantity and quality were analyzed using a bibliometric analysis study of the past ten years. This is the first study reporting global trends related to bone tissue scaffold using alginate, gelatine and hydroxyapatite. Throughout this paper, the main keywords ‘bone tissue’ and ‘scaffold’, together with sub keywords ‘alginate’, ‘gelatine’, and ‘hydroxyapatite’ refer to the bibliometric analysis. The most apparent findings arising from this analysis are:

- A total of 7446 publications with the keywords ‘‘bone tissue” and scaffold were found, while 1767 (alginate), 185 (gelatine), 5658 (hydroxyapatite) papers with the specific sub keywords were determined from 2009 to 2019.
- Article type comes into prominence as the dominant category in terms of the type of publication.
- China and the United States are the most productive countries, according to the total publication criteria.
- While Boccaccini Aldo Roberto, from Germany, is the most productive author in terms of publication number, Ramakrishna Seeram is the most productive author considering the average citations per article, with 63.74 points.
- The most preferred keywords are bone tissue engineering, scaffold and bone regeneration for alginate.
- Over 94.14% of the publications were published in English.
- Material Science and Engineering C takes the leading place, with 89 (alginate), 12 (gelatine), and 288 (hydroxyapatite) publications and a 10.2 impact factor.
- In moving the related research forward, a better interpretation of bibliometric analysis needs to evolve. More information on this type of research would facilitate the production of a greater degree of precision on this subject.

Despite the numerous advantages of combining alginate, gelatine, and hydroxyapatite, various challenges remain when it comes to applications where further improvements are necessary. 3D bioprinting, clinical outcomes, scaffold architecture, and regenerative medicine approach can be considered as future perspectives in the field of bone tissue regeneration applications. In addition, it is expected that the number of studies relating alginate, gelatine, and hydroxyapatite to these applications will rise further in the next decade.

## Figures and Tables

**Table 1:** Number of publications based on the Scopus database using keywords alginate, gelatine, hydroxyapatite, scaffold, bone tissue.

Keywords	Publications (1969–2019)	Publications (2009–2019)
Alginate	35,572	23,038
Gelatine	3853	1544
Hydroxyapatite	58,489	31,349
Scaffold	133,383	107,064
Bone Tissue	34,198	22,270
Alginate + Scaffold + Bone Tissue	315	284
Gelatine + Scaffold + Bone Tissue	24	23
Hydroxyapatite + Scaffold + Bone Tissue	2920	2404
Alginate + Hydroxyapatite + Scaffold + Bone Tissue	105	94
Gelatine + Hydroxyapatite + Scaffold + Bone Tissue	13	13
Alginate + Gelatine + Scaffold + Bone Tissue	4	4
Alginate + Gelatine + Hydroxyapatite + Scaffold + Bone Tissue	2	2

**Table 2:** Types of published documents (2009–2019).

	Alginate	Gelatine	Hydroxyapatite	Alg	Gel	Hyd
Article	1329	135	4526	75.20%	73.00%	80.00%
Conference Paper	40	6	342	2.30%	3.20%	6.04%
Review	282	32	516	16.00%	17.30%	9.12%
Book Chapter	113	12	242	6.40%	6.49%	4.28%
Other	3	0	32	0.00%	0.00%	0.01%
Total	1767	185	5658			

**Table 3:** Scientific publications by country (2009–2019).

	Number of Publication			Percentage %			Number of Citation			H-Index (Rank)		
Country	Alg	Gel	Hyd	Alg	Gel	Hyd	Alg	Gel	Hyd	Alg	Gel	Hyd
China	511	40	1617	28.92	21.62	28.58	9166	858	21,910	54 (2)	16 (2)	82 (2)
United States	338	12	973	19.13	6.49	17.20	13,097	412	26,727	67 (1)	9 (5)	101 (1)
India	181	18	389	10.24	9.73	6.88	5208	782	8714	41 (4)	11 (4)	58 (4)
South Korea	144	11	423	8.15	5.95	7.48	3747	427	9621	34 (5)	8 (6)	56 (5)
Iran	131	13	404	7.41	7.03	7.14	2953	225	6688	32 (6)	9 (5)	45 (7)
Germany	114	26	349	6.45	14.05	6.17	5067	863	10,273	43 (3)	17 (1)	63 (3)
Italy	92	21	352	5.21	11.35	6.22	3541	512	9190	31 (7)	12 (3)	55 (6)
United Kingdom	81	6	272	4.58	3.24	4.81	4312	148	8804	34 (5)	5 (8)	55 (6)
Portugal	52	4	128	2.94	2.16	2.26	2353	97	4433	27 (8)	4 (9)	42 (8)
Turkey	51	9	128	2.89	4.86	2.26	830	106	2006	18 (9)	6 (7)	27 (9)
Total	1767	185	5658	100	100	100						

Alg = Alginate; Gel = Gelatine; Hyd = Hydroxyapatite.

**Table 4:** Authors with the highest number of publications (2009–2019).

	Number of Publication			Total Citation	Citation/Article	H-Index
Authors	Alginate	Gelatine	Hydroxyapatite			
Boccaccini Aldo Roberto	50	11	111	44,249	35.74	92
Reis Rui Luis	21	2	55	45,911	37.30	99
Xu Hockin H.K.	19	0	23	10,777	61.23	67
Chang Jiang	18	2	39	21,026	44.93	80
Selvamurugan Nagarajan	18	2	29	7704	56.23	47
Wu Chengtie	17	3	42	12,183	48.35	64
Venkatesan Jayachandran	16	2	25	4274	34.19	34
Kim Se Kwon	15	2	24	30,725	43.15	92
Zhao Liang	15	0	16	1304	27.17	20
Ramakrishna Seeram	15	1	43	83,183	63.74	137
Roether Judith A.	14	3	21	5132	42.77	36
Weir Michael D.	14	0	18	2001	12.91	23
Gelinsky Michael	13	5	23	6007	24.03	43
Azami Mahmoud	12	1	27	1827	25.38	28
Weng Jie	12	0	21	6546	24.80	42

Data retrieved on December 2020.

**Table 5:** Most frequent keywords used in publication titles (2009–2019).

Alginate		Gelatine		Hydroxyapatite	
Word	Frequency	Word	Frequency	Word	Frequency
Bone	1189	Saffold	104	Bone	3655
Tissue	847	Bone	103	Scaffold	3340
Engineering	697	Tissue	81	Tissue	2394
Scaffold	981	Engineering	61	Engineering	1996
Cell	359	Composite	28	Composite	1048
Regeneration	329	Hydroxyapatite	27	Regeneration	799
Composite	252	Regeneration	26	Hydroxyapatite	714
Stem	220	Cell	24	Stem	628
Porous	143	Porous	21	Porous	600
3D	138	Bioactive	20	Phosphate	454
Calcium	133	3D	18	Vitro	432
Phosphate	130	Characterization	16	Bioactive	413
Mesenshymal	126	Glass	15	Calcium	397
Differentiation	108	Vitro	14	Properties	388
Vitro	102	Gelatine	7	Mesenchymal	379

**Table 6:** Top ten publishing journals (2009–2019).

	Material Science & Engineering C	Journal of Biomedical Materials Research Part A	Acta Biomaterialia	International Journal of Biological Macromolecules	Biomaterials	RSC Advances	Colloids and Surfaces B Biointerfaces	Carbohydrate Polymers	Chinese Journal of Tissue Engineering Research	Journal of Material Science Materials in Medicine
Alginate	89	79	62	58	46	42	34	33	31	31
Gelatine	12	11	7	8	2	3	1	2	2	3
Hydroxyapatite	288	267	198	95	127	93	71	48	109	153
CiteScore (2019)	10.2	6.6	11.8	6.9	18.7	6.5	7.1	11.7	0.1	4.9

**Table 7:** Most productive institutions.

Institutions	Number of Publications			Total Citation			H-Index		
	Alg	Gel	Hyd	Alg	Gel	Hyd	Alg	Gel	Hyd
Sichuan University	61	4	167	1551	127	3755	26	3	36
Chinese Academy of Science	53	3	148	1524	165	4319	22	3	40
Friedrich Alexander-Universitat Erlangen	51	13	115	3027	304	4526	25	10	40
Amirkabir University of Technology	36	8	92	877	132	1972	19	6	30
National University of Singapore	23	2	82	946	46	3134	15	2	32
Shanghai JiaoTong University	26	4	77	551	60	1964	13	3	26
Central South University	12	0	67	313	0	1347	7	0	23
Tehran University of Medical Science	22	0	65	624	0	1486	12	0	26
Universidade do Minho	24	3	63	1549	83	2803	16	3	31
South China University of Technology	24	0	63	555	0	1421	14	0	26

Alg = Alginate; Gel = Gelatine; Hyd = Hydroxyapatite.

## Data Availability

The data presented in this study are available on request from the corresponding author.
